# Supplementary material for: Investigation of allele-specific expression in citrus hybrids reveals the association between siRNA-mediated de novo methylation and high expression in citrus genomes
Source: Hortic Res. 2026 May 4;13(10):uhag183. doi: 10.1093/hr/uhag183 (PMC13610574; doi:10.1093/hr/uhag183)
Supplement: Supplementary file 1 [file web_material_uhag183.zip › Supp_Figures&TablesDIOPet al R3.docx]

**Supplemental Figures and Tables**

***Figure S1. Anchoring of the haplotype assemblies to the consensus Citrus genetic map.***

*Each ribbon connects a genomic scaffold from the haplotype assembly (left) to its corresponding position in one of the nine linkage groups (right). (****A****) Haplotype derived from C. reticulata. (****B****) Haplotype derived from C. australasica. The strong one-to-one correspondence and limited cross-linking support the structural accuracy of the assemblies.*

***Figure S2. Validation of RNA-seq analyses by RT-qPCR.***

*Analyses of transcripts accumulation of five different expressed genes in the parental and the hybrid lines according to the RNA-seq analyses (left graphic) or by RT-qPCR using C. reticulata-specific primers (middle praphic) or C. australasica-specific primers (right graphics). RT-qPCR of genes presented in the panel* ***B-D*** *were normalized according the housekeeping gene encoding UBIQUITIN (panel* ***A****; 001g001500).*

***Figure S3. REVIGO TreeMaps of enriched Biological Process (GO) terms among differentially expressed genes (DEGs) in the hybrid.***

***(A)*** *GO terms enriched among genes upregulated from the C. australasica haplotype. Major categories include ubiquitin-dependent protein catabolic process, protein catabolic/metabolic process, translation, RNA modification and transport, and stress and defence responses.* ***(B)*** *GO terms enriched among genes upregulated from the C. reticulata haplotype. Enriched categories include photosynthesis, protein phosphorylation, RNA processing, DNA replication, NADP biosynthetic process, and other central metabolic and transcriptional functions, indicating a predominant maternal contribution to core cellular activities.*

***Figure S4. Exclusive allelic expression in the hybrid.***

*Pie-chart diagram showing the origin of the allele exclusively expressed by the C. australasica haplotype (right) or the C. reticulata haplotype (left) in the hybrid. These genes are then separated in four categories: those that are only expressed in the hybrid and not in the parental lines (Hybrid-specific; blue), those that are also expressed in C. australasica (C. aus; green) or in C. reticulata (C. ret; orange) parental lines and those that are expressed in both parents (C. aus + C. ret; beige colour).*

***Figure S5. Genomic distribution of DNA methylation in the hybrid.***

*The distribution of the three DNA methylation patterns (CG, CHG and CHH) is shown across all haplotype resolved hybrid C. aus (****A****) or hybrid C. ret. (****B****) chromosomes.*

***Figure S6. WGBS validation using BS-PCR and Sanger sequencing***

*To validate the methylation profiles obtained by WGBS, we designed primers at five different genomic location, performed bisulfite treatment and amplified selected promoter regions using specific primers. Browser screenshot displays the data obtained using WGBS and RNA-seq data, separating CHH and CG methylation. The black square corresponds to the genomic location amplified by PCR, cloned and sequenced using Sanger technic. Methylation analyses were then performed using CYMATE (*[*www.cymate.org*](http://www.cymate.org)*). As expected, unmethylated promoter regions in WGBS are also unmethylated when analysed by BS-PCR (****A****,* ***C****), while methylated promoter regions accumulate methylation in CHH context only (****D****), or at both CG and CHH context (****B****,* ***E****).*

***Figure S7: Sensitivity analysis of promoter methylation thresholds.***

*(A–B) Hybrid haplotypes: the same analysis as in Figure 3C was performed using alternative methylation thresholds:(A) CHH ≥ 15%, CG ≥ 25%; (B) CHH ≥ 10%, CG ≥ 20%. (C) Cross-species analysis: the same analysis as in Figure 7 was performed using CHH ≥ 15% and CG ≥ 25%.*

***Figure S8. screenshot of IGV browser.***

*IGV screenshot examples showing the distribution of small RNA clusters, mRNA accumulation, and CG or CHH methylation accumulation along 4 genomic loci in the hyb C. ret (****A, C****) or Hyb C. aus (****B, D****). In these 4 cases, the presence of siRNA clusters and mCHH in the promoter regions is not incompatible with gene expression.*

***Figure S9. SiRNA clusters in the hybrid.***

*Venn diagram showing the overlap of unique 24-nt siRNA sequences identified from clusters annotated independently in each haplotype (C. reticulata and C. australasica). About one third of 24-nt siRNA sequences (1630) are shared between the two haplotypes, while the remaining sequences are unique to one or the other: 1612 being specific to C. australasica haplotype (left, green), and 1695 being specific to the C. reticulata (right, orange) haplotype.*

***Figure S10: Sensitivity analysis of expression threshold. The same analysis as in Fig. 6A–B was performed using a more stringent expression threshold (TPM > 1). (A) Genes with expression (TPM > 1) and promoter siRNA clusters. (B) Genes with low or no expression (TPM ≤ 1) and promoter siRNA clusters. The overall pattern remains consistent with the main analysis.***

***Figure S11. Genome-wide epigenomic patterns and overlap with expression in the parental lines.***

*(****A****) Dot-plot alignment between the maternal haplotype of the hybrid C. ret and CITRE reference.* ***(B****) Dot-plot alignment between the paternal haplotype hybrid C. aus and the C. australasica reference genome. These plots illustrate overall synteny and structural rearrangements between each haplotype of the hybrid and the closest available reference genome from the corresponding parental line. Diagonal lines indicate conserved homologous regions, while off-diagonal signals highlight structural variations. (****C–D****) Circos plots representing genome-wide profiles of DNA methylation, gene density, and 24-nt siRNA*

*clusters across the nine chromosomes of the two parental lines C. reticulata (****C****) and C. australasica (****D****).*

***Figure S12. Specific analyses of siRNA–CHH methylation–gene expression modules in the C. ret, C. aus and in the hybrid.***

*Metagene plots displaying 24-nt siRNA accumulation (top), CHH methylation (middle panel) and gene expression of siRNA–CHH methylation–gene modules with expressed genes (left panel) or silent genes (right panel) in C. reticulata (****A****), in C. australasica (****B****), or in the hybrid with an haplotype resolution (****C-D****). Metagene plots displays the accumulation from -2kb to +2kb of the Transcription start site (TSS).*

***Figure S13.*** ***Venn diagrams showing the overlap between siRNA presence and methylation categories in parental genomes.***

*Each plot displays the number of genes in a given methylation class (grey) and those with 24-nt siRNA clusters in their promoters (yellow) in C. reticulata (****A****) or in C. australasica (****B****). Overlaps represent genes with both features. Separate diagrams are shown for each methylation category.*

***Figure S14. DNA methylation associating with the promoter of DEG in the hybrid***

*Violin plots displaying the distribution of DEG genes for each haplotype according to the type of methylation accumulating in their promoter regions. CHH > 20%; CG > 40%.*

***Figure S15. Relative expression of RdDM members in the hybrid and the parent***

*Analyses of the relative expression levels of several major homologs of the RdDM in the hybrid haplotypes and in the parental lines. Data were extracted from the RNA-seq and are presented as table (****A****) and in the graphic* ***(B****). TPM =* Transcripts Per Kilobase Million

***Table S1. Summary of RNA-seq data processing and mapping.***

*This table presents, for each replicate, the total number of raw reads, the number of reads retained after trimming, the number of mapped reads, and the total mapping rate (%). All samples exhibit high mapping rates (>98%), confirming the high quality of the RNA-seq libraries and the reliability of gene expression quantification in hybrid and parental genotypes.*

***Table S2. Mapping statistics of WGBS data for hybrid and parental samples.***

*For each biological replicate, the table reports the number of paired-end reads obtained, the number of uniquely aligned pairs, the mapping efficiency (%), the duplication rate (%), the number of deduplicated reads, the estimated genome size used for mapping (in megabases), and the average sequencing depth (X). The samples include the hybrid mapped to both parental haplotypes (Hyb_C.ret and Hyb_C.aus), as well as the two parental species (C. reticulata and C. australasica).*

***Table S3. List and sequences of the primer used in this study.***

***Table S4. Effect size summary for promoter methylation and gene expression in hybrid haplotypes.***

| origin | group1 | group2 | | N group1 | | N group2 | | Median group1 | | Median group2 | | Delta median | | Cliffs delta | | P value | | P adj | | P adj_label | |
| --- | --- | --- | --- | --- | --- | --- | --- | --- | --- | --- | --- | --- | --- | --- | --- | --- | --- | --- | --- | --- | --- |
| C_ret | No meth | CG & CHH | 20106 | | 3069 | | 2.354 | | 0.758 | | -1.595 | | -0.146 | | 3.03e-39 | | 3.03e-39 | | <2.2e-16 | |  |
| C_ret | No meth | CG only | 20106 | | 6533 | | 2.354 | | 0.658 | | -1.695 | | -0.183 | | 3.79e-111 | | 1.14e-110 | | <2.2e-16 | |  |
| C_ret | No meth | CHH only | 20106 | | 1272 | | 2.354 | | 4.218 | | 1.864 | | 0.236 | | 1.08e-45 | | 1.62e-45 | | <2.2e-16 | |  |
| Hyb_C_aus | No meth | CG & CHH | 20944 | | 3020 | | 2.247 | | 0.085 | | -2.162 | | -0.202 | | 6.41e-74 | | 9.61e-74 | | <2.2e-16 | |  |
| Hyb_C_aus | No meth | CG only | 20944 | | 5754 | | 2.247 | | 0.391 | | -1.856 | | -0.173 | | 1.15e-92 | | 3.46e-92 | | <2.2e-16 | |  |
| Hyb_C_aus | No meth | CHH only | 20944 | | 1262 | | 2.247 | | 3.903 | | 1.655 | | 0.193 | | 2.98e-31 | | 2.98e-31 | | <2.2e-16 | |  |
| Hyb_C_ret | No meth | CG & CHH | 20773 | | 3031 | | 2.275 | | 0.172 | | -2.102 | | -0.179 | | 3.43e-58 | | 5.14e-58 | | <2.2e-16 | |  |
| Hyb_C_ret | No meth | CG only | 20773 | | 5864 | | 2.275 | | 0.376 | | -1.899 | | -0.186 | | 8.00e-108 | | 2.40e-107 | | <2.2e-16 | |  |
| Hyb_C_ret | No meth | CHH only | 20773 | | 1312 | | 2.275 | | 4.019 | | 1.744 | | 0.224 | | 1.01e-42 | | 1.01e-42 | | <2.2e-16 | |  |
| C_aus | No meth | CG & CHH | 22155 | | 1795 | | 2.001 | | 0.674 | | -1.327 | | -0.147 | | 2.43e-25 | | 3.64e-25 | | <2.2e-16 | |  |
| C_aus | No meth | CG only | 22155 | | 6370 | | 2.001 | | 0.676 | | -1.325 | | -0.151 | | 8.97e-77 | | 2.69e-76 | | <2.2e-16 | |  |
| C_aus | No meth | CHH only | 22155 | | 660 | | 2.001 | | 3.475 | | 1.474 | | 0.176 | | 1.11e-14 | | 1.11e-14 | | 1.11e-14 | |  |

*For each comparison, we report the number of genes in each group (n), median expression values (log₂(mean TPM + 1)), median shifts (Δmed), Cliff’s delta, and adjusted p-values (Benjamini–Hochberg correction). Promoters were classified into four categories based on methylation levels (No methylation, CG only, CHH only, CG & CHH; thresholds: CHH ≥ 20%, CG ≥ 40%). Positive Δmed values indicate higher expression in group2 relative to group1.*

***Table S5. Effect size summary for promoter siRNA presence and gene expression.***

| origin | group1 | group2 | n_group1 | n_group2 | median_group1 | median_group2 | delta_median | cliffs_delta | p_value |
| --- | --- | --- | --- | --- | --- | --- | --- | --- | --- |
| C_ret | All genes | With siRNA in promoter | 30980 | 1513 | 2.019 | 3.446 | 1.427 | 0.127 | 3.34e-17 |
| C_ret | With siRNA in promoter | Without siRNA in promoter | 1513 | 29467 | 3.446 | 1.947 | -1.499 | -0.134 | 8.12e-19 |
| Hyb_C_aus | All genes | With siRNA in promoter | 30980 | 752 | 1.873 | 2.453 | 0.580 | 0.072 | 5.75e-04 |
| Hyb_C_aus | With siRNA in promoter | Without siRNA in promoter | 752 | 30228 | 2.453 | 1.855 | -0.599 | -0.074 | 4.17e-04 |
| Hyb_C_ret | All genes | With siRNA in promoter | 30980 | 765 | 1.871 | 3.053 | 1.182 | 0.095 | 5.79e-06 |
| Hyb_C_ret | With siRNA in promoter | Without siRNA in promoter | 765 | 30215 | 3.053 | 1.846 | -1.207 | -0.097 | 3.34e-06 |
| C_aus | All genes | With siRNA in promoter | 30980 | 1996 | 1.724 | 3.098 | 1.374 | 0.133 | 9.69e-24 |
| C_aus | With siRNA in promoter | Without siRNA in promoter | 1996 | 28984 | 3.098 | 1.645 | -1.453 | -0.142 | 8.51e-27 |

*For each comparison, we report the number of genes in each group (n), median expression values (log₂(mean TPM + 1)), median shifts (Δmed), Cliff’s delta, and adjusted p-values (Benjamini–Hochberg correction). Genes were classified based on the presence or absence of 24-nt siRNA clusters in promoter regions (1 kb upstream of the transcription start site). Comparisons involving “All genes” are shown relative to the genome-wide background. Positive Δmed values indicate higher expression in group2 relative to group1.*

***Table S6. Effect size summary for promoter methylation and gene expression across citrus species.***

| origin | group1 | group2 | n_group1 | n_group2 | Median  group1 | Median  group2 | delta median | Cliffs delta | p value | p adj | p adj_label |
| --- | --- | --- | --- | --- | --- | --- | --- | --- | --- | --- | --- |
| C_reshni | No methylation | CG & CHH | 21390 | 2901 | 2.726 | 2.322 | -0.404 | -0.088 | 1.13e-14 | 1.13e-14 | 1.13e-14 |
| C_reshni | No methylation | CG only | 21390 | 9512 | 2.726 | 0.226 | -2.500 | -0.277 | 0 | 0 | <2.2e-16 |
| C_reshni | No methylation | CHH only | 21390 | 907 | 2.726 | 4.252 | 1.526 | 0.242 | 2.28e-35 | 3.42e-35 | <2.2e-16 |
| C_trifoliata | No methylation | CG & CHH | 18760 | 1800 | 3.473 | 3.474 | 0.001 | -0.058 | 4.91e-05 | 4.91e-05 | 4.91e-05 |
| C_trifoliata | No methylation | CG only | 18760 | 4197 | 3.473 | 1.288 | -2.185 | -0.282 | 4.89e-181 | 1.47e-180 | <2.2e-16 |
| C_trifoliata | No methylation | CHH only | 18760 | 780 | 3.473 | 4.255 | 0.782 | 0.185 | 1.41e-18 | 2.12e-18 | <2.2e-16 |
| C_lemon | No methylation | CG & CHH | 17774 | 1359 | 2.877 | 3.538 | 0.661 | 0.082 | 4.81e-07 | 4.81e-07 | 4.81e-07 |
| C_lemon | No methylation | CG only | 17774 | 5766 | 2.877 | 1.396 | -1.481 | -0.186 | 3.02e-101 | 9.07e-101 | <2.2e-16 |
| C_lemon | No methylation | CHH only | 17774 | 783 | 2.877 | 3.991 | 1.114 | 0.219 | 1.72e-25 | 2.57e-25 | <2.2e-16 |
| C_sinensis | No methylation | CG & CHH | 17515 | 1219 | 3.435 | 2.498 | -0.937 | -0.150 | 1.31e-18 | 1.97e-18 | <2.2e-16 |
| C_sinensis | No methylation | CG only | 17515 | 8752 | 3.435 | 0.512 | -2.922 | -0.334 | 0 | 0 | <2.2e-16 |
| C_sinensis | No methylation | CHH only | 17515 | 321 | 3.435 | 4.599 | 1.164 | 0.245 | 4.73e-14 | 4.73e-14 | 4.73e-14 |

***For each comparison, we report the number of genes in each group (n), median expression values (log₂(mean TPM + 1)), median shifts (Δmed), Cliff’s delta, and adjusted p-values (Benjamini–Hochberg correction). Promoters were classified into four categories based on methylation levels (No methylation, CG only, CHH only, CG & CHH; thresholds: CHH ≥ 20%, CG ≥ 40%). Positive Δmed values indicate higher expression in group2 relative to group1.***
