# Supplementary material for: Investigation of allele-specific expression in citrus hybrids reveals the association between siRNA-mediated de novo methylation and high expression in citrus genomes
Source: Hortic Res. 2026 May 4;13(10):uhag183. doi: 10.1093/hr/uhag183 (PMC13610574; doi:10.1093/hr/uhag183)
Supplement: Supplementary file 1 [file web_material_uhag183.zip › Supp_Figures&TablesDIOPet al R3.pdf]

Supplemental Figures and Tables

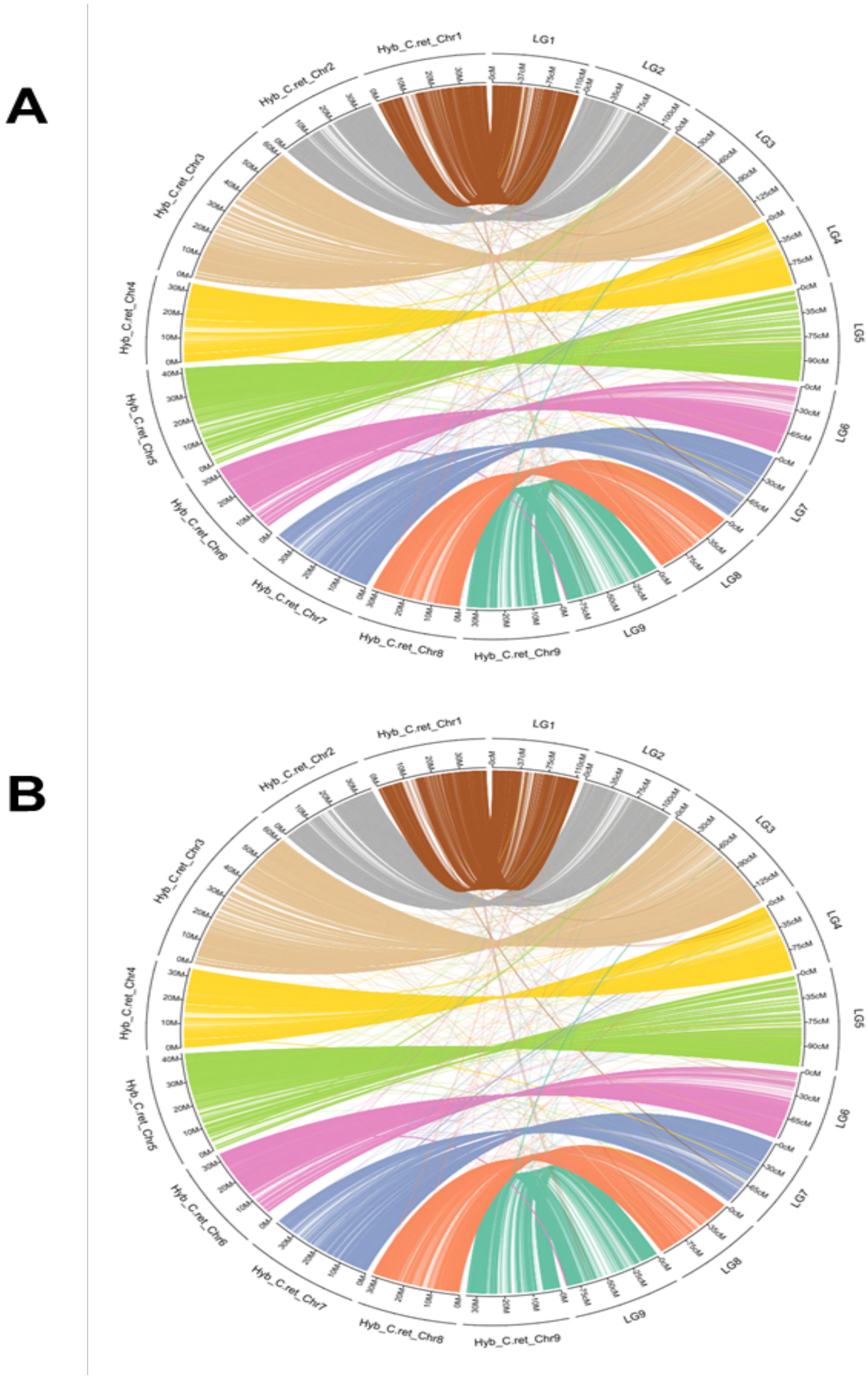

Figure S1. Anchoring of the haplotype assemblies to the consensus Citrus genetic map. *Each ribbon connects a genomic scaffold from the haplotype assembly (left) to its corresponding position in one of the nine linkage groups (right). (**A**) Haplotype derived from C. reticulata. (**B**) Haplotype derived from C. australasica. The strong one-to-one correspondence and limited cross-linking support the structural accuracy of the assemblies.*

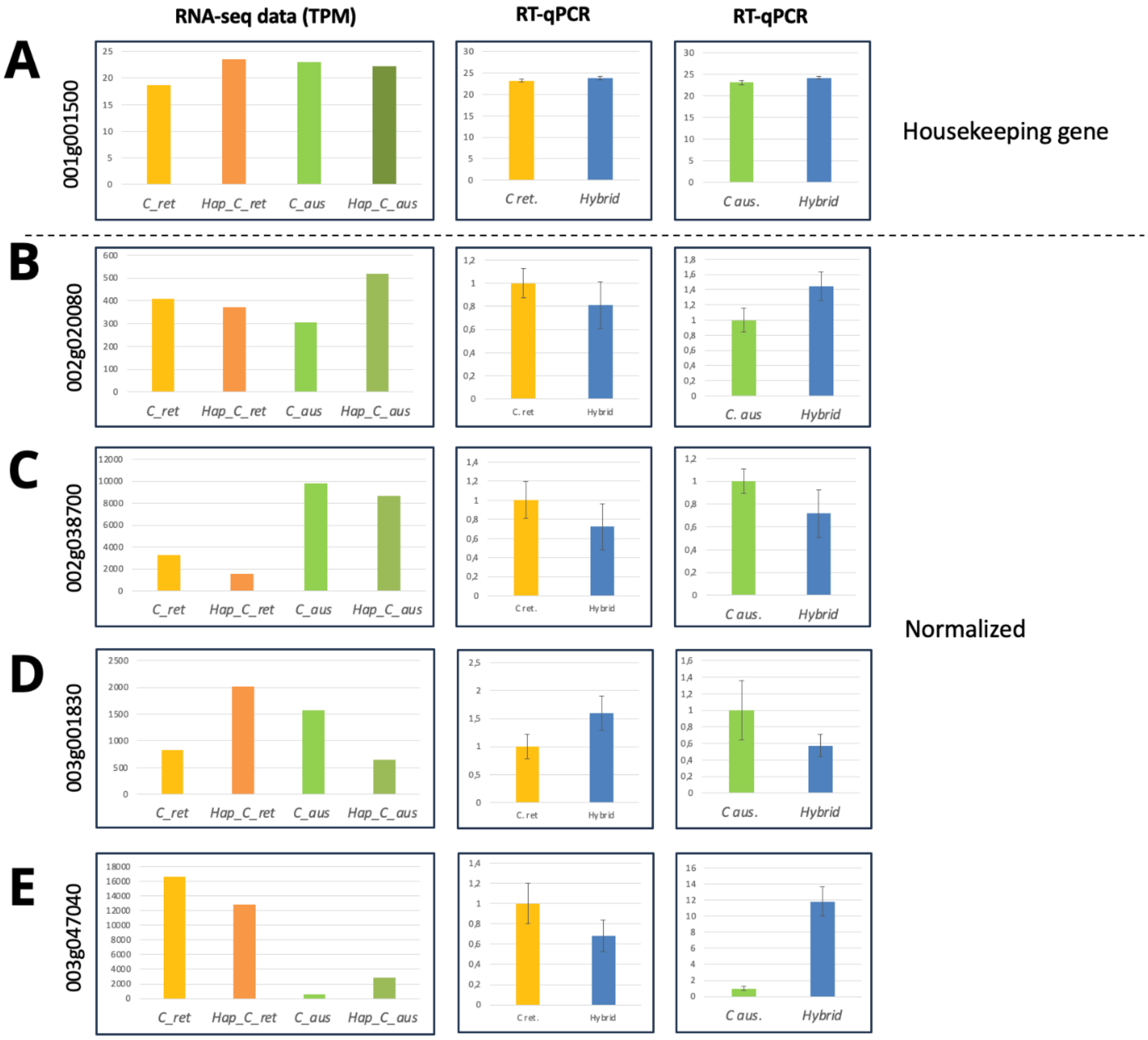

Figure S2. Validation of RNA-seq analyses by RT-qPCR.

*Analyses of transcripts accumulation of five different expressed genes in the parental and the hybrid lines according to the RNA-seq analyses (left graphic) or by RT-qPCR using C. reticulata-specific primers (middle praphic) or C. australasica-specific primers (right graphics). RT-qPCR of genes presented in the panel **B-D** were normalized according the housekeeping gene encoding UBIQUITIN (panel **A**; 001g001500).*

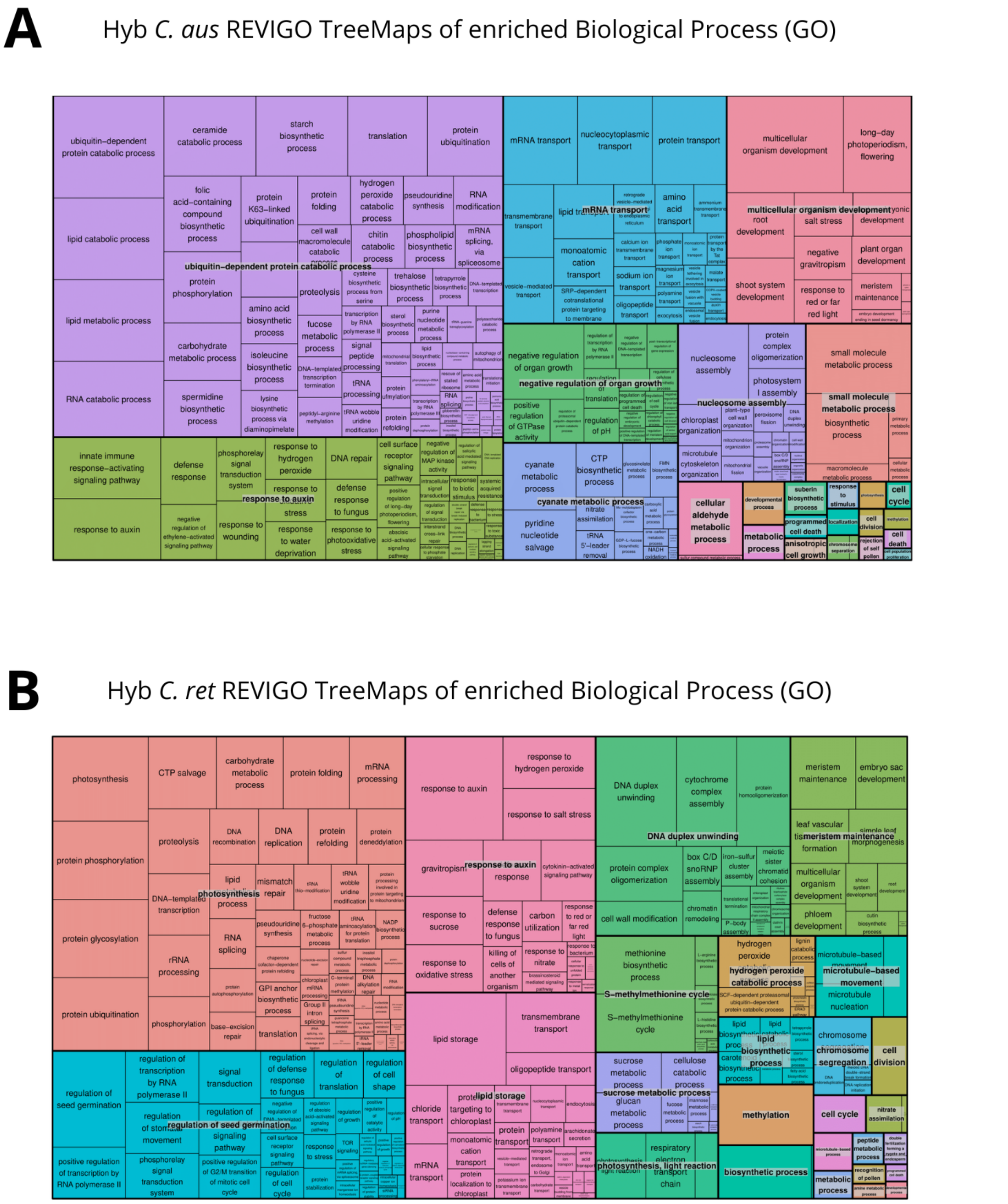

Figure S3. REVIGO TreeMaps of enriched Biological Process (GO) terms among differentially expressed genes (DEGs) in the hybrid.

(A) *GO terms enriched among genes upregulated from the C. australasica haplotype. Major categories include ubiquitin-dependent protein catabolic process, protein catabolic/metabolic process, translation, RNA modification and transport, and stress and defence responses.* ***(B)*** *GO terms enriched among genes upregulated from the C. reticulata haplotype. Enriched categories include photosynthesis, protein phosphorylation, RNA processing, DNA replication, NADP biosynthetic process, and other central metabolic and transcriptional functions, indicating a predominant maternal contribution to core cellular activities.*

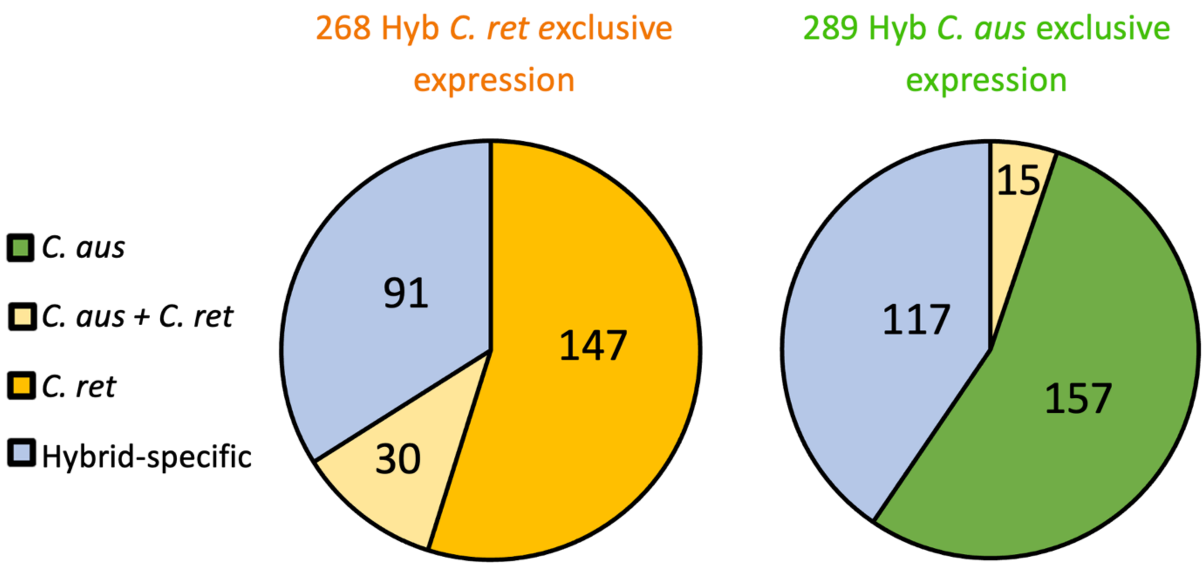

Figure S4. Exclusive allelic expression in the hybrid.

Pie-chart diagram showing the origin of the allele exclusively expressed by the C. australasica haplotype (right) or the C. reticulata haplotype (left) in the hybrid. These genes are then separated in four categories: those that are only expressed in the hybrid and not in the parental lines (Hybrid-specific; blue), those that are also expressed in C. australasica (C. aus; green) or in C. reticulata (C. ret; orange) parental lines and those that are expressed in both parents (C. aus + C. ret; beige colour).

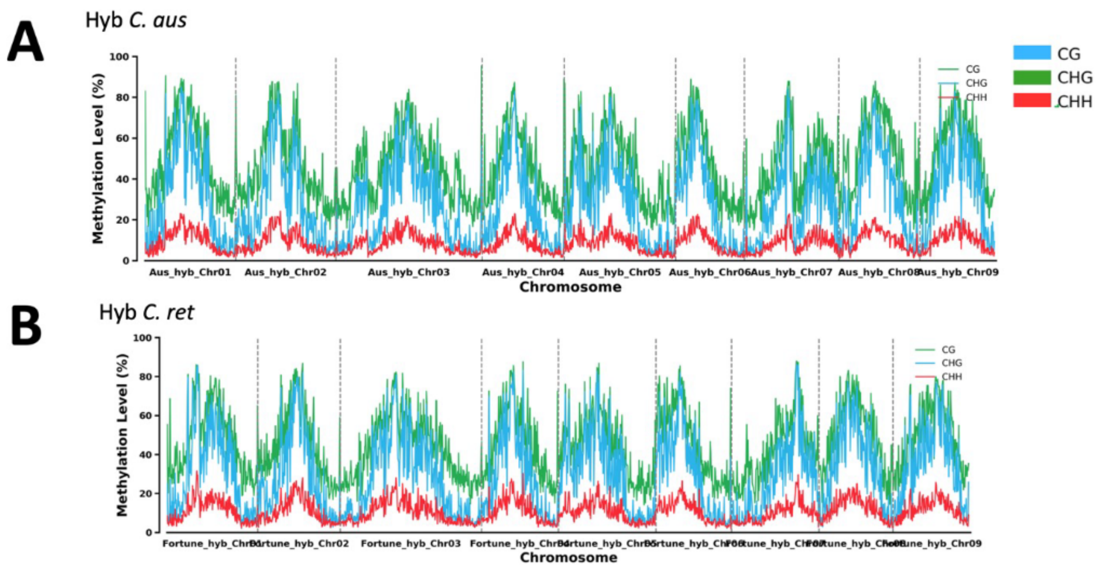

Figure S5. Genomic distribution of DNA methylation in the hybrid.

*The distribution of the three DNA methylation patterns (CG, CHG and CHH) is shown across all haplotype resolved hybrid C. aus (**A**) or hybrid C. ret. (**B**) chromosomes.*

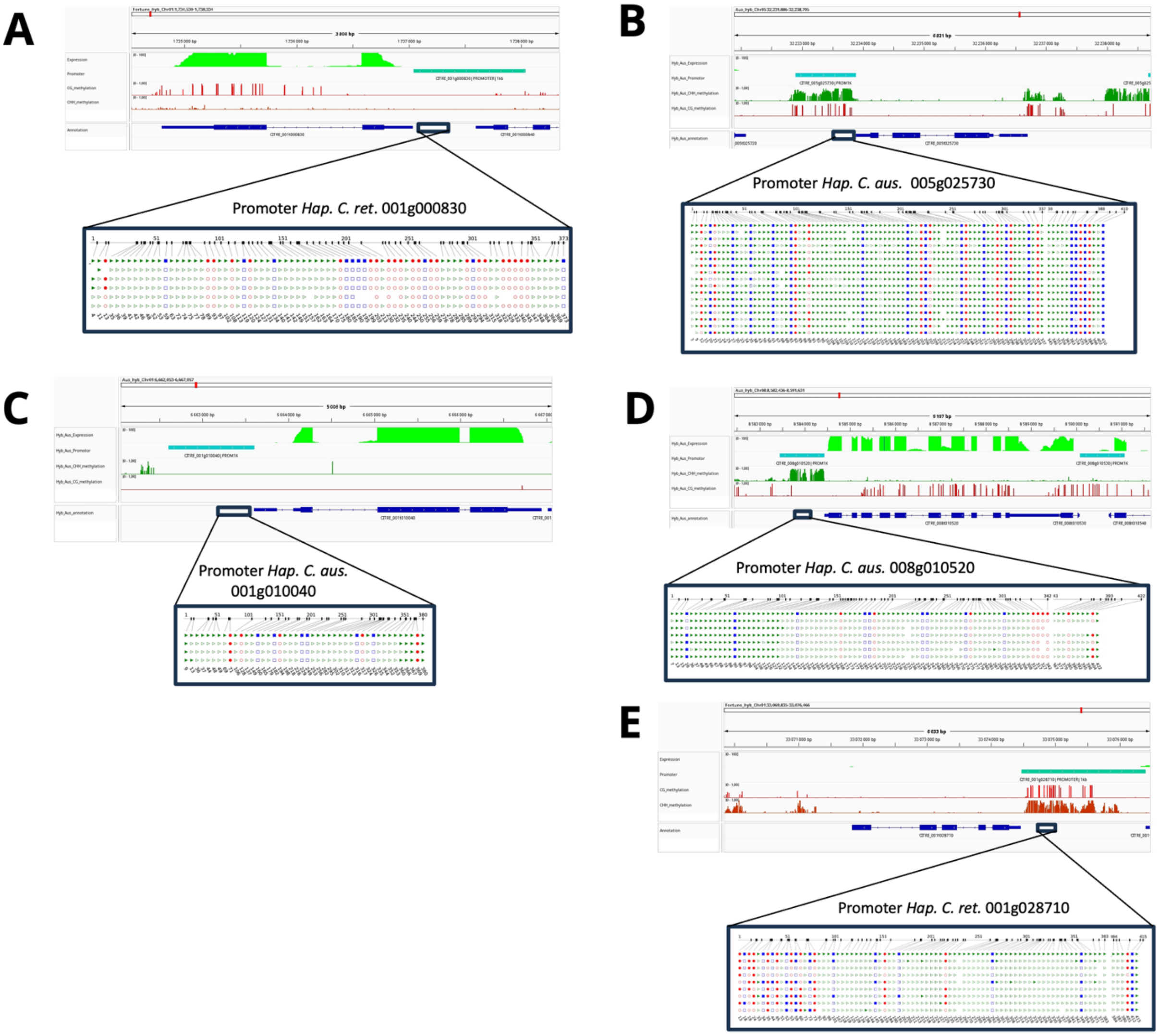

Figure S6. WGBS validation using BS-PCR and Sanger sequencing

*To validate the methylation profiles obtained by WGBS, we designed primers at five different genomic location, performed bisulfite treatment and amplified selected promoter regions using specific primers. Browser screenshot displays the data obtained using WGBS and RNA-seq data, separating CHH and CG methylation. The black square corresponds to the genomic location amplified by PCR, cloned and sequenced using Sanger technic. Methylation analyses were then performed using CYMATE (www.cymate.org). As expected, unmethylated promoter regions in WGBS are also unmethylated when analysed by BS-PCR (**A**, **C**), while methylated promoter regions accumulate methylation in CHH context only (**D**), or at both CG and CHH context (**B**, **E**).*

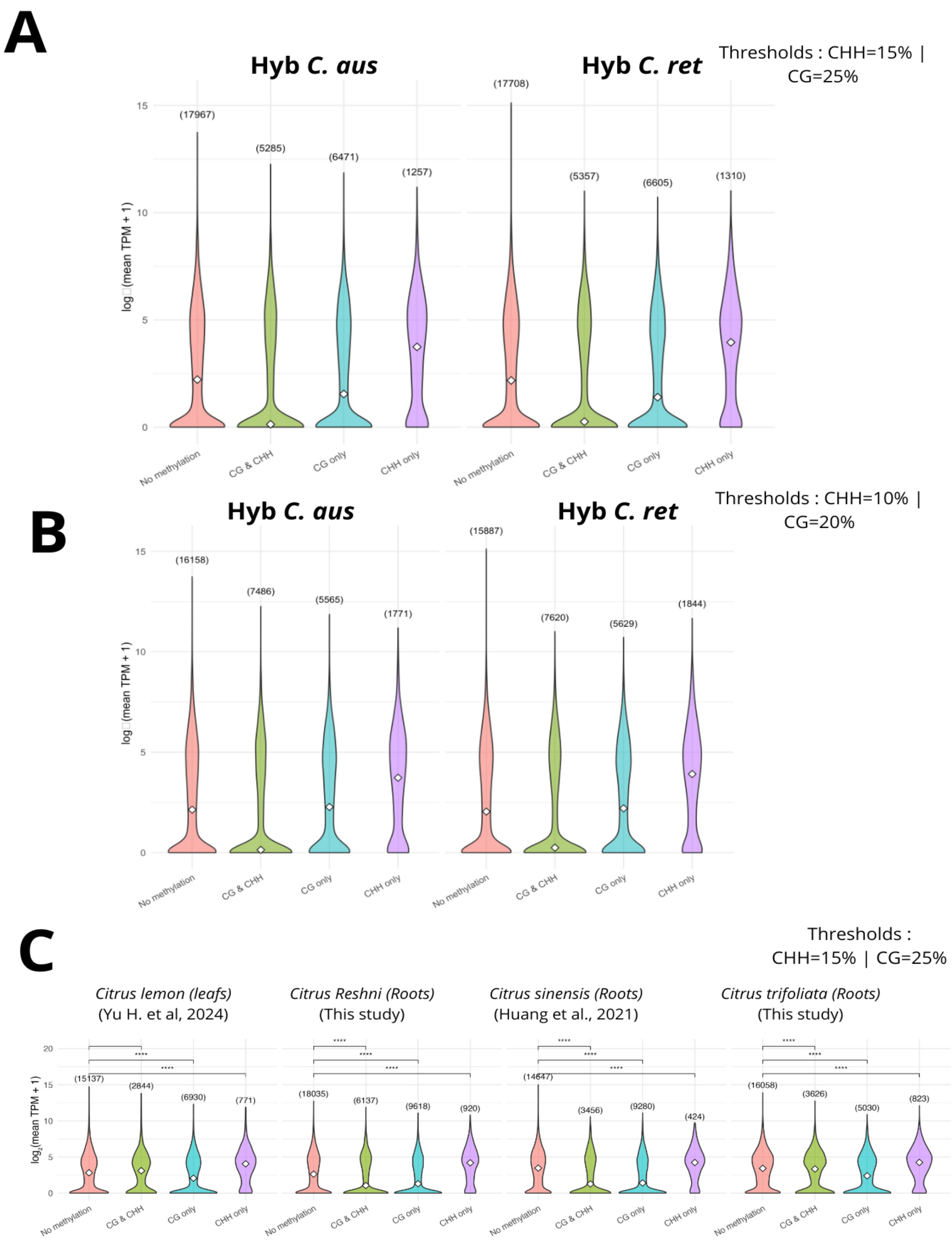

Figure S7: Sensitivity analysis of promoter methylation thresholds.

(A–B) Hybrid haplotypes: the same analysis as in Figure 3C was performed using alternative methylation thresholds:(A) CHH ≥ 15%, CG ≥ 25%; (B) CHH ≥ 10%, CG ≥ 20%. (C) Cross-species analysis: the same analysis as in Figure 7 was performed using CHH ≥ 15% and CG ≥ 25%.

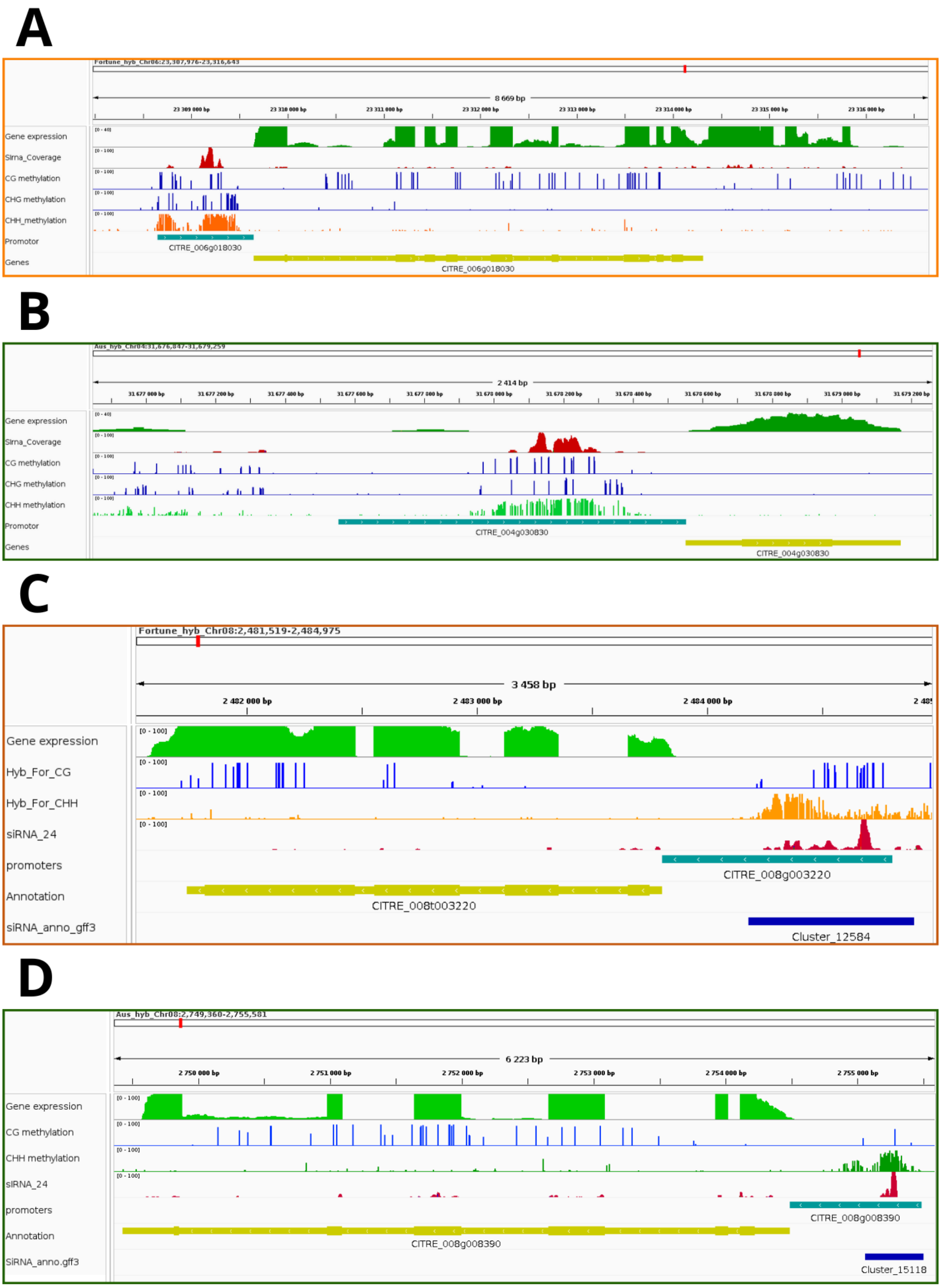

Figure S8. screenshot of IGV browser.

*IGV screenshot examples showing the distribution of small RNA clusters, mRNA accumulation, and CG or CHH methylation accumulation along 4 genomic loci in the hyb C. ret (**A, C**) or Hyb C. aus (**B, D**). In these 4 cases, the presence of siRNA clusters and mCHH in the promoter regions is not incompatible with gene expression.*

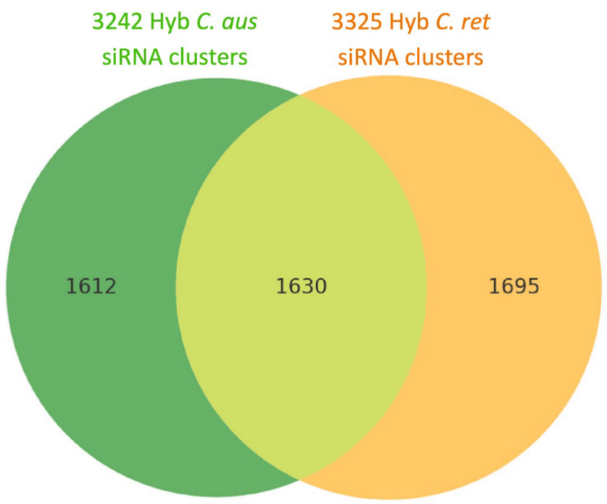

Figure S9. SiRNA clusters in the hybrid.

Venn diagram showing the overlap of unique 24-nt siRNA sequences identified from clusters annotated independently in each haplotype (C. reticulata and C. australasica). About one third of 24-nt siRNA sequences (1630) are shared between the two haplotypes, while the remaining sequences are unique to one or the other: 1612 being specific to C. australasica haplotype (left, green), and 1695 being specific to the C. reticulata (right, orange) haplotype.

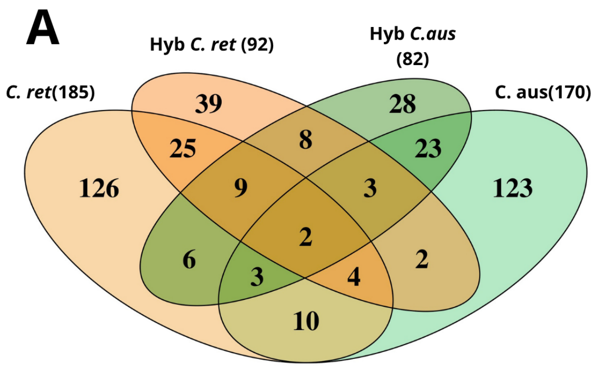

Figure S10: Sensitivity analysis of expression threshold. The same analysis as in Fig. 6A–B was performed using a more stringent expression threshold (TPM > 1). (A) Genes with expression (TPM > 1) and promoter siRNA clusters. (B) Genes with low or no expression (TPM ≤ 1) and promoter siRNA clusters. The overall pattern remains consistent with the main analysis.

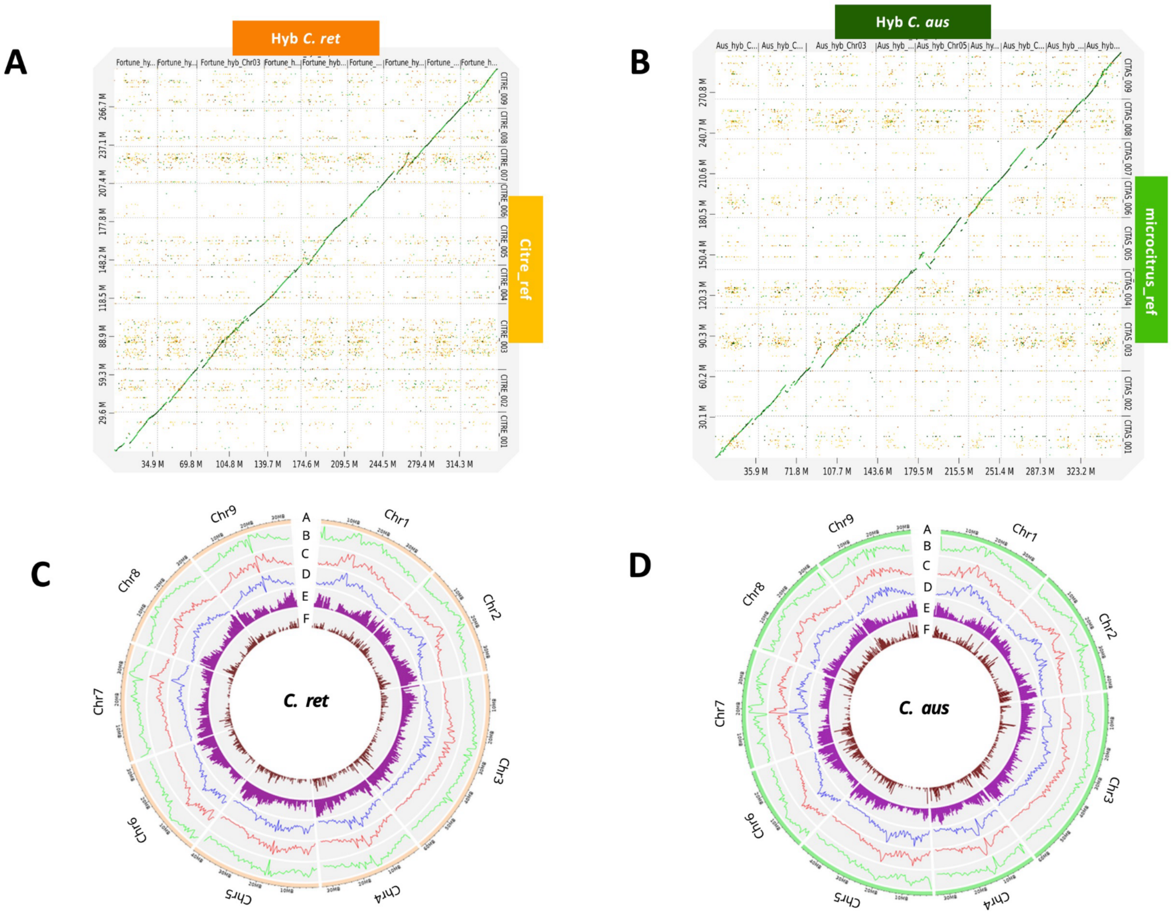

Figure S11. Genome-wide epigenomic patterns and overlap with expression in the parental lines.

*(**A**) Dot-plot alignment between the maternal haplotype of the hybrid C. ret and CITRE reference. (**B**) Dot-plot alignment between the paternal haplotype hybrid C. aus and the C. australasica reference genome. These plots illustrate overall synteny and structural rearrangements between each haplotype of the hybrid and the closest available reference genome from the corresponding parental line. Diagonal lines indicate conserved homologous regions, while off-diagonal signals highlight structural variations. (**C–D**) Circos plots representing genome-wide profiles of DNA methylation, gene density, and 24-nt siRNA clusters across the nine chromosomes of the two parental lines C. reticulata (**C**) and C. australasica (**D**).*

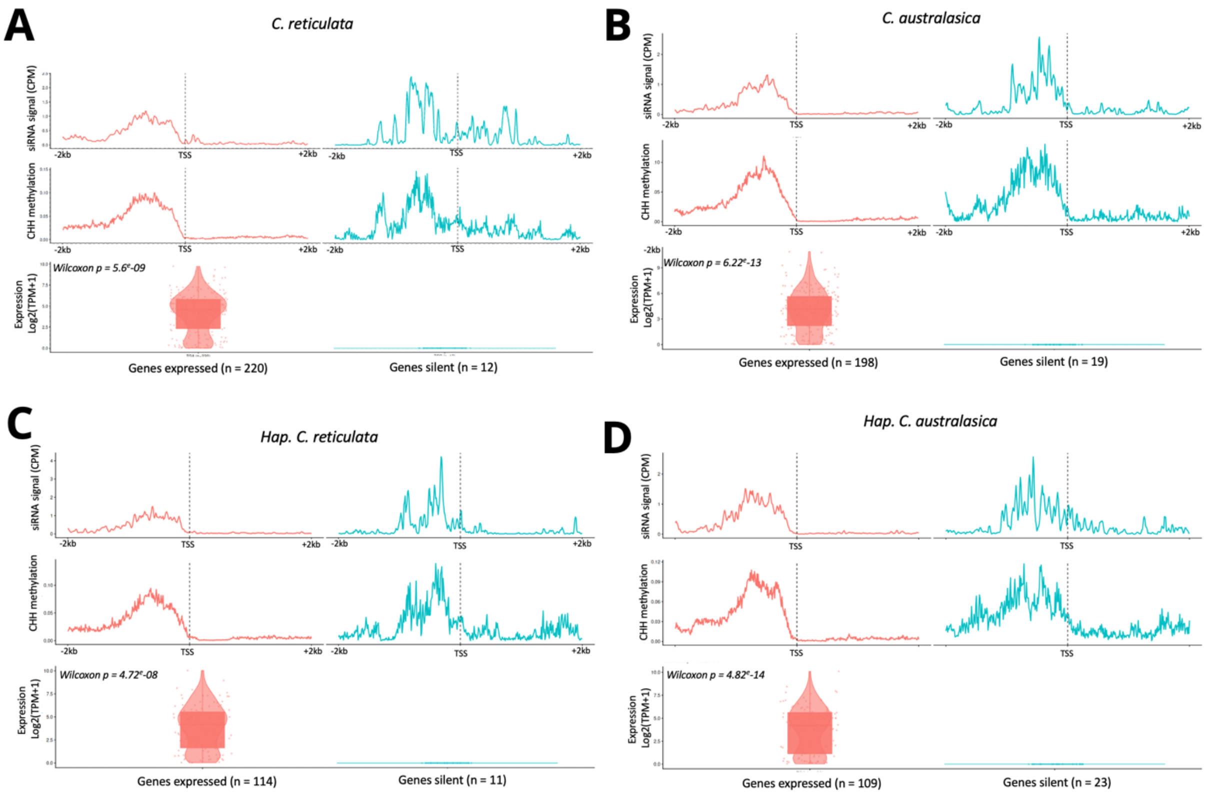

Figure S12. Specific analyses of siRNA–CHH methylation–gene expression modules in the C. ret, C. aus and in the hybrid.

*Metagene plots displaying 24-nt siRNA accumulation (top), CHH methylation (middle panel) and gene expression of siRNA–CHH methylation–gene modules with expressed genes (left panel) or silent genes (right panel) in C. reticulata (**A**), in C. australasica (**B**), or in the hybrid with an haplotype resolution (**C-D**). Metagene plots displays the accumulation from -2kb to +2kb of the Transcription start site (TSS).*

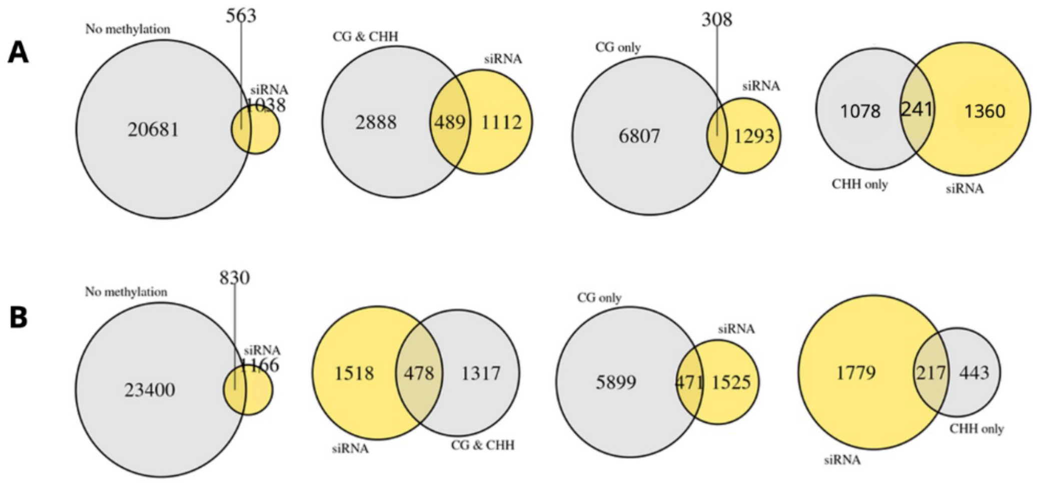

Figure S13. Venn diagrams showing the overlap between siRNA presence and methylation categories in parental genomes.

*Each plot displays the number of genes in a given methylation class (grey) and those with 24-nt siRNA clusters in their promoters (yellow) in C. reticulata (**A**) or in C. australasica (**B**). Overlaps represent genes with both features. Separate diagrams are shown for each methylation category.*

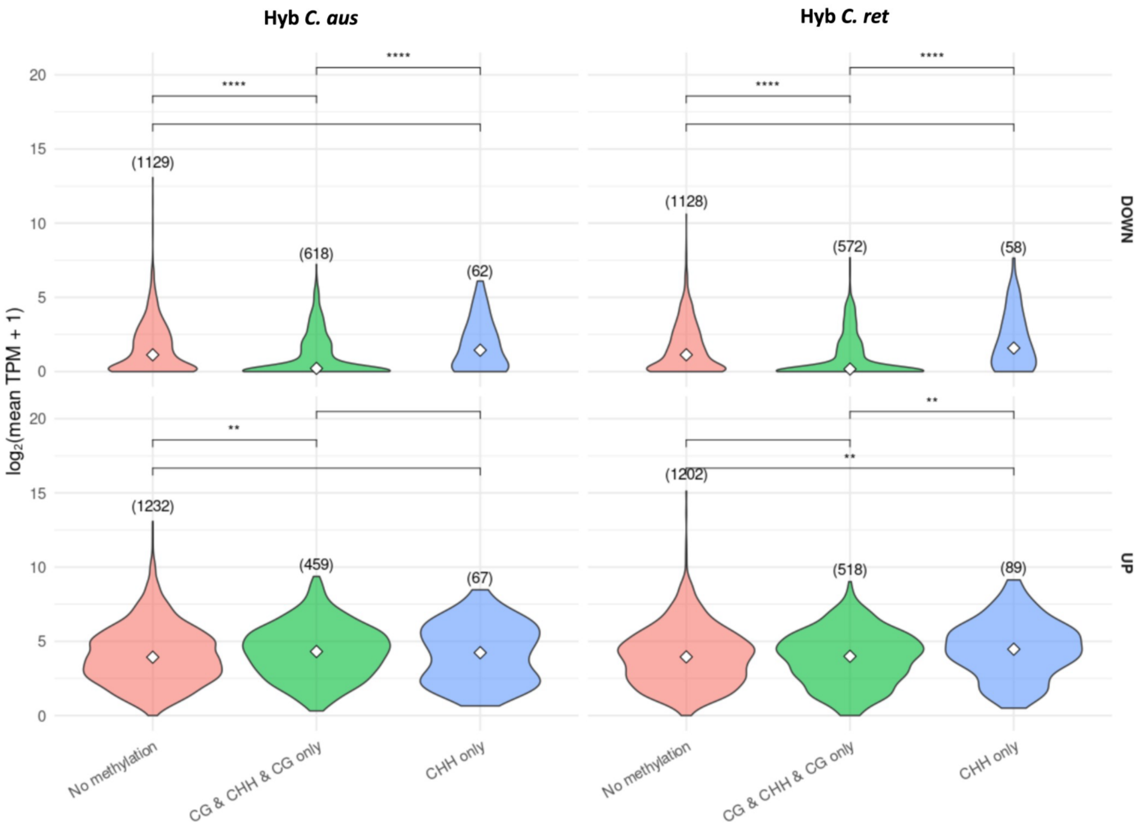

Figure S14. DNA methylation associating with the promoter of DEG in the hybrid

Violin plots displaying the distribution of DEG genes for each haplotype according to the type of methylation accumulating in their promoter regions. CHH > 20%; CG > 40%.

A

Genes	Citrus_gene_id	*C. Reticulata*	*Hap. C. ret.*	*C. australasi*	*Hap. C. aus.*
**NRPD1**	CITRE_003g011380	4,767334	4,29413733	2,158684	1,429438
**NRPE1**	CITRE_008g018100	11,3111357	10,2296567	13,6122613	15,0771083
**DRM2**	CITRE_002g008120	27,0843727	41,0615447	25,4039753	30,5621017
**AGO4**	CITRE_002g006780	164,122676	155,941335	130,161471	124,150246
**AGO6**	CITRE_002g020700	7,006687	7,03087667	4,41796067	3,92255767
**SUVH4/KYP**	CITRE_002g012110	13,5418107	19,1116547	9,62826167	10,1865453
**SUVH6**	CITRE_005g013450	39,1862233	39,4222197	40,0289423	41,050663
**MET1**	CITRE_007g009140	1,88593033	1,90298567	1,343297	3,34992833
**CMT3**	CITRE_002g001610	0,94181633	1,84237067	1,16343533	0,95102667
**CMT2**	CITRE_005g014740	0,978142	2,56786967	16,1104	18,615868
**DDM1**	CITRE_007g003580	1,08242133	1,89476467	1,29368567	1,48642333

B

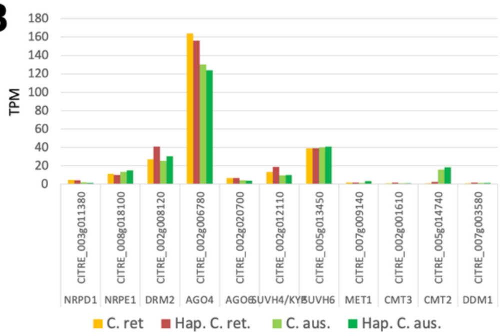

Figure S15. Relative expression of RdDM members in the hybrid and the parent

*Analyses of the relative expression levels of several major homologs of the RdDM in the hybrid haplotypes and in the parental lines. Data were extracted from the RNA-seq and are presented as table (**A**) and in the graphic **(B)**. TPM =* Transcripts Per Kilobase Million

Table S1. Summary of RNA-seq data processing and mapping.

**Sample**	**Raw Reads**	**Trimmed Reads**	**Mapped Reads**	**Total Mapped (%)**
Hyb_C.ret_rep1	31,570,252	29,619,476	29,179,963	98.51
Hyb_C.ret_rep2	31,149,835	29,499,214	29,085,818	98.57
Hyb_C.ret_rep3	31,411,499	29,720,267	29,294,474	98.57
*C. reticulata*_rep1	31,136,378	29,435,073	28,976,206	98.44
*C. reticulata*_rep2	32,938,492	31,231,204	30,636,695	98.09
*C. reticulata*_rep3	31,448,963	29,651,782	29,120,932	98.21
*C. australasica*_rep1	28,905,022	27,272,835	26,792,030	98.24
*C. australasica*_rep2	31,215,753	29,470,075	28,936,627	98.19
*C. australasica*_rep3	30,971,028	29,478,682	28,978,279	98.30
Hyb_C.aus_rep1	31,570,252	29,619,476	29,254,611	98.77
Hyb_C.aus_rep1	31,149,835	29,499,214	29,164,128	98.86
Hyb_C.aus_rep1	31,411,499	29,720,267	29,367,248	98.81

This table presents, for each replicate, the total number of raw reads, the number of reads retained after trimming, the number of mapped reads, and the total mapping rate (%). All samples exhibit high mapping rates (>98%), confirming the high quality of the RNA-seq libraries and the reliability of gene expression quantification in hybrid and parental genotypes.

Table S2. Mapping statistics of WGBS data for hybrid and parental samples.

Samples	Sequence pairs	Number of paired-end alignments with a unique best hit	Mapping Efficiency (%)	Duplication Rate (%)	Deduplicated Leftover Sequences	Genome Size (Mb)	Average Depth (X)
Hyb_C.aus_rep1	59471997	45299369	76.2	21.17	35707603	359	25.56
Hyb_C.aus_rep2	56997431	43090193	75.6	23.2	33092958	359	24.5
Hyb_C.aus_rep3	52490561	40235003	76.7	22.88	31029064	359	22.56
Hyb_C.ret_rep1	59471997	44722620	75.2	21.75	34997498	349	25.56
Hyb_C.ret_rep2	56997431	42539674	74.6	23.69	32461335	349	24.5
Hyb_C.ret_rep3	52490561	39753625	75.7	23.55	30389785	349	22.56
*C. reticulata*_rep1	54120503	43591838	80.5	23.67	33274917	349	23.96
*C. reticulata*_rep2	53416001	41156597	77.0	22.69	31818808	349	22.82
*C. reticulata*_rep3	50019636	38564496	77.1	18.48	31436564	349	22.67
*C. australasica*_rep1	53564928	41617819	77.7	26.86	30441058	359	21.67
*C. australasica*_rep2	54127489	41896425	77.4	26.90	30627038	359	21.81
*C. australasica*_rep3	53798201	41649738	77.4	25.70	30946374	359	21.97

For each biological replicate, the table reports the number of paired-end reads obtained, the number of uniquely aligned pairs, the mapping efficiency (%), the duplication rate (%), the number of deduplicated reads, the estimated genome size used for mapping (in megabases), and the average sequencing depth (X). The samples include the hybrid mapped to both parental haplotypes (Hyb_C.ret and Hyb_C.aus), as well as the two parental species (C. reticulata and C. australasica).

Table S3. List and sequences of the primer used in this study.

Orientation	Targeted region / Primer name	primer sequence	experiment
**For**	Fortune_hyb_Chr01:1834900-1835400(-)CITRE_001g000830	AATYAAATTTYTYGAGAGTGAAGAGAAG	BS-PCR
**Rev**	Fortune_hyb_Chr01:1834900-1835400(-)CITRE_001g000830	CRTCATTRAATTCTCTRTTACATRAATT	BS-PCR
**For**	Aus_hyb_Chr02:25606838-25607338(-)CITRE_001g010040	YATTTATTTCTYTYTTAAGTTAAAGAGGTGATT	BS-PCR
**Rev**	Aus_hyb_Chr02:25606838-25607338(-)CITRE_001g010040	TARTCTCATCCACRARRATTTTRTAAAT	BS-PCR
**For**	Aus_hyb_Chr05:598460-598960(+)CITRE_005g025730	ACTTYYATYGTTTGYTYAAATGGYGG	BS-PCR
**Rev**	Aus_hyb_Chr05:598460-598960(+)CITRE_005g025730	AACCARTAAAAATRACAAARATACCCAAA	BS-PCR
**For**	Aus_hyb_Chr02:39307299-39307799(+)CITRE_008g010520	ATGGTAYTTGTGYACAAAAATGGGG	BS-PCR
**Rev**	Aus_hyb_Chr02:39307299-39307799(+)CITRE_008g010520	TARTCTTTACACCCATTACCRCAAAARAA	BS-PCR
**For**	Fortune_hyb_Chr06:15304575-15305075(+)CITRE_001g028710	ATATTTTGAGTGYGGGTYYGGTAYGGG	BS-PCR
**Rev**	Fortune_hyb_Chr06:15304575-15305075(+)CITRE_001g028710	CAARCARTTTCATCAACRATATTTTCAA	BS-PCR
**For**	1g001500a_F	AGCATTCAGCAAATCTAGCTAGG	RT-qPCR
**Rev**	1g001500ab_R	GTGACATCCGATCTCAGTTGTC	RT-qPCR
**For**	1g001500b_F	CAGCATTCAGCAAATCTAGCTAGT	RT-qPCR
**For**	2g020080b_F	TACATATTTATCCTGGCAGTGCA	RT-qPCR
**Rev**	2g020080b_R	CCACGCCAGCTCAACTGCG	RT-qPCR
**For**	2g020080a_F	TACATATTTATCCTGGCAGTGCG	RT-qPCR
**Rev**	2g020080a_R	CCACGCCAGCTCAACTGCA	RT-qPCR
**For**	2g038700a_F	CATGCGTAAAACCGGGAGAC	RT-qPCR
**Rev**	2g038700a_R	CAGTGTCCCAACCGTAGTCA	RT-qPCR
**For**	2g038700b_F	CATGCGTAAAACCGGGAGCA	RT-qPCR
**Rev**	2g038700b_R	CTGTGTCCCAACCGTAGTCG	RT-qPCR
**For**	3g001830a_F	CCAATACACGACCGCTGGAC	RT-qPCR
**Rev**	3g001830a_R	GGGCTTACCCGTGTTGTAGT	RT-qPCR
**For**	3g001830b_F	CCAATACACGACCGCTGGAA	RT-qPCR
**Rev**	3g001830b_R	GGGCTTACCCGTGTTGTAGC	RT-qPCR
**For**	3g047040a_F	AAGCACAAGGGAGAGCACCAG	RT-qPCR
**Rev**	3g047040a_R	GGTGAACATCAGTAGTGCCGG	RT-qPCR
**For**	3g047040b_F	AAGCACAAGGGTGAGCACCAT	RT-qPCR
**Rev**	3g047040b_R	ATGGTGAACATCAGTAGTACCGA	RT-qPCR

Table S4. Effect size summary for promoter methylation and gene expression in hybrid haplotypes.

origin	group1	group2	N group1	N group2	Median group1	Median group2	Delta median	Cliffs delta	P value	P adj	P adj_label
**C_ret**	No meth	CG & CHH	20106	3069	2.354	0.758	-1.595	-0.146	3.03 e-39	3.03 e-39	<2.2 e-16
**C_ret**	No meth	CG only	20106	6533	2.354	0.658	-1.695	-0.183	3.79 e-111	1.14 e-110	<2.2 e-16
**C_ret**	No meth	CHH only	20106	1272	2.354	4.218	1.864	0.236	1.08 e-45	1.62 e-45	<2.2 e-16
**Hyb_C_aus**	No meth	CG & CHH	20944	3020	2.247	0.085	-2.162	-0.202	6.41 e-74	9.61 e-74	<2.2 e-16
**Hyb_C_aus**	No meth	CG only	20944	5754	2.247	0.391	-1.856	-0.173	1.15 e-92	3.46 e-92	<2.2 e-16
**Hyb_C_aus**	No meth	CHH only	20944	1262	2.247	3.903	1.655	0.193	2.98 e-31	2.98 e-31	<2.2 e-16
**Hyb_C_ret**	No meth	CG & CHH	20773	3031	2.275	0.172	-2.102	-0.179	3.43 e-58	5.14 e-58	<2.2 e-16
**Hyb_C_ret**	No meth	CG only	20773	5864	2.275	0.376	-1.899	-0.186	8.00 e-108	2.40 e-107	<2.2 e-16
**Hyb_C_ret**	No meth	CHH only	20773	1312	2.275	4.019	1.744	0.224	1.01 e-42	1.01 e-42	<2.2 e-16
**C_aus**	No meth	CG & CHH	22155	1795	2.001	0.674	-1.327	-0.147	2.43 e-25	3.64 e-25	<2.2 e-16
**C_aus**	No meth	CG only	22155	6370	2.001	0.676	-1.325	-0.151	8.97 e-77	2.69 e-76	<2.2 e-16
**C_aus**	No meth	CHH only	22155	660	2.001	3.475	1.474	0.176	1.11 e-14	1.11 e-14	1.11 e-14

For each comparison, we report the number of genes in each group (n), median expression values (log_2(mean TPM + 1)), median shifts (Δmed), Cliff's delta, and adjusted p-values (Benjamini–Hochberg correction). Promoters were classified into four categories based on methylation levels (No methylation, CG only, CHH only, CG & CHH; thresholds: CHH ≥ 20%, CG ≥ 40%). Positive Δmed values indicate higher expression in group2 relative to group1.

Table S5. Effect size summary for promoter siRNA presence and gene expression.

origin	group1	group2	n_group1	n_group2	median_group1	median_group2	delta_median	cliffs_delta	p_value
C_ret	All genes	With siRNA in promoter	30980	1513	2.019	3.446	1.427	0.127	3.34e-17
C_ret	With siRNA in promoter	Without siRNA in promoter	1513	29467	3.446	1.947	-1.499	-0.134	8.12e-19
Hyb_C_aus	All genes	With siRNA in promoter	30980	752	1.873	2.453	0.580	0.072	5.75e-04
Hyb_C_aus	With siRNA in promoter	Without siRNA in promoter	752	30228	2.453	1.855	-0.599	-0.074	4.17e-04
Hyb_C_ret	All genes	With siRNA in promoter	30980	765	1.871	3.053	1.182	0.095	5.79e-06
Hyb_C_ret	With siRNA in promoter	Without siRNA in promoter	765	30215	3.053	1.846	-1.207	-0.097	3.34e-06
C_aus	All genes	With siRNA in promoter	30980	1996	1.724	3.098	1.374	0.133	9.69e-24
C_aus	With siRNA in promoter	Without siRNA in promoter	1996	28984	3.098	1.645	-1.453	-0.142	8.51e-27

For each comparison, we report the number of genes in each group (n), median expression values (log_2(mean TPM + 1)), median shifts (Δmed), Cliff's delta, and adjusted p-values (Benjamini–Hochberg correction). Genes were classified based on the presence or absence of 24-nt siRNA clusters in promoter regions (1 kb upstream of the transcription start site). Comparisons involving "All genes" are shown relative to the genome-wide background. Positive Δmed values indicate higher expression in group2 relative to group1.

Table S6. Effect size summary for promoter methylation and gene expression across citrus species.

origin	group1	group2	n_group1	n_group2	Median group1	Median group2	delta median	Cliffs delta	p value	p adj	p adj_label
**C_reshni**	No methylation	CG & CHH	21390	2901	2.726	2.322	-0.404	-0.088	1.13e-14	1.13e-14	1.13e-14
**C_reshni**	No methylation	CG only	21390	9512	2.726	0.226	-2.500	-0.277	0	0	<2.2e-16
**C_reshni**	No methylation	CHH only	21390	907	2.726	4.252	1.526	0.242	2.28e-35	3.42e-35	<2.2e-16
**C_trifoliata**	No methylation	CG & CHH	18760	1800	3.473	3.474	0.001	-0.058	4.91e-05	4.91e-05	4.91e-05
**C_trifoliata**	No methylation	CG only	18760	4197	3.473	1.288	-2.185	-0.282	4.89e-181	1.47e-180	<2.2e-16
**C_trifoliata**	No methylation	CHH only	18760	780	3.473	4.255	0.782	0.185	1.41e-18	2.12e-18	<2.2e-16
**C_lemon**	No methylation	CG & CHH	17774	1359	2.877	3.538	0.661	0.082	4.81e-07	4.81e-07	4.81e-07
**C_lemon**	No methylation	CG only	17774	5766	2.877	1.396	-1.481	-0.186	3.02e-101	9.07e-101	<2.2e-16
**C_lemon**	No methylation	CHH only	17774	783	2.877	3.991	1.114	0.219	1.72e-25	2.57e-25	<2.2e-16
**C_sinensis**	No methylation	CG & CHH	17515	1219	3.435	2.498	-0.937	-0.150	1.31e-18	1.97e-18	<2.2e-16
**C_sinensis**	No methylation	CG only	17515	8752	3.435	0.512	-2.922	-0.334	0	0	<2.2e-16
**C_sinensis**	No methylation	CHH only	17515	321	3.435	4.599	1.164	0.245	4.73e-14	4.73e-14	4.73e-14

For each comparison, we report the number of genes in each group (n), median expression values (log_2(mean TPM + 1)), median shifts (Δmed), Cliff's delta, and adjusted p-values (Benjamini–Hochberg correction). Promoters were classified into four categories based on methylation levels (No methylation, CG only, CHH only, CG & CHH; thresholds: CHH ≥ 20%, CG ≥ 40%). Positive Δmed values indicate higher expression in group2 relative to group1.
